# A review of short-term weather impacts on honey production

**DOI:** 10.1007/s00484-024-02824-0

**Published:** 2024-12-07

**Authors:** Csilla Vincze, Ádám Leelőssy, Edit Zajácz, Róbert Mészáros

**Affiliations:** 1 Institute of Geography and Earth Sciences, Department of Meteorology, ELTE Eötvös Loránd University, Budapest, Hungary; 2 Institute for Farm Animal Gene Conservation, Department of Apiculture and Bee Biology, National Centre for Biodiversity and Gene Conservation, Gödöllő, Hungary

**Keywords:** Honeybee, Bee foraging, Weather sensitivity, Honey production, Bee flight activity

## Abstract

Beekeeping is an exceptionally weather-sensitive agricultural field. Honey production and pollination services depend on the complex interaction of plants and bees, both of which are impacted by short-term weather changes. In this review, classical and recent research is collected to provide an overview on short-term atmospheric factors influencing honey production, and the optimal and critical weather conditions for bee activity. Bee flight can be directly obstructed by precipitation, wind, extreme temperatures and also air pollution. Bees generally fly within a temperature range of 10–40 °C, with optimal foraging efficiency occurring between 20 and 30 °C. Wind speeds exceeding 1.6–6.7 m/s can reduce foraging efficiency. Additionally, bee activity is significantly correlated with temperature, relative humidity and solar radiation, factors which influence nectar production. Optimal conditions for nectar collection typically occur in the morning and early afternoon hours with mild and moist weather. The diurnal nectar collection habit of bees adjusts to the nectar production of individual plant species. Extreme weather occurring in the sensitive hours is noticeable both in the nectar production of plants and in the activity of bees, thus in the honey yield. Understanding the impact of weather on honey bees is crucial in the management and planning of honey production. This review highlights the importance of studying these interactions to better adapt beekeeping practices to changing environmental conditions.

**Graphical Abstract:**

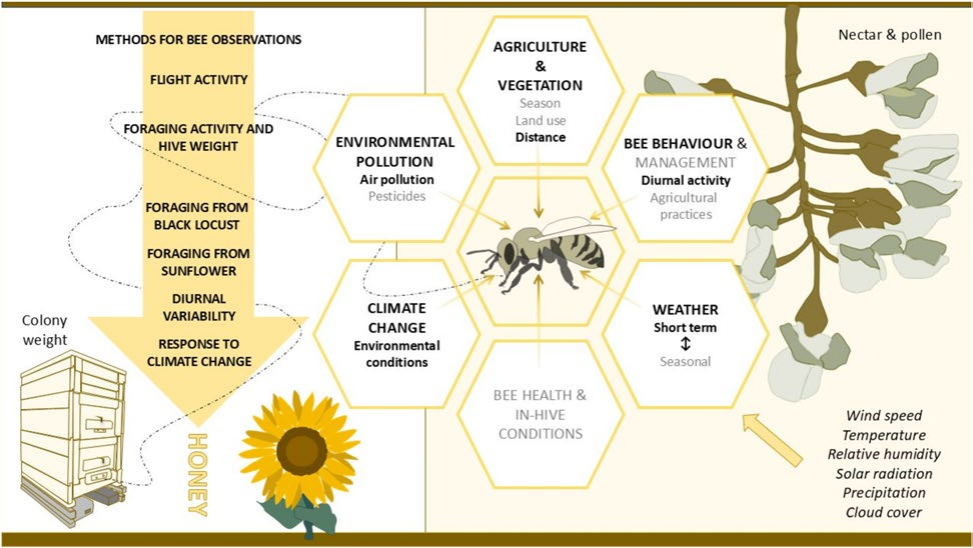

## Introduction

Beekeeping is one of the most weather-sensitive forms of agriculture. Honey bees (*Apis mellifera*) play a crucial role in ecosystems and society, pollinating about 35% of human food (Synge 1947; Klein et al. 2007; Genersch 2010; Potts et al. 2010; Rader et al. 2013; Tarczay and Feiler 2017; FAO 2018; Otieno et al. 2020).

It has long been accepted that one of the most important factors in beekeeping is the weather (Mace 1912). Research has shown that the beehive activity (Southwick and Moritz 1987; Human et al. 2006), honey yield and quality (Mace 1912; Robertson et al. 2010), intention to collect (Santas 1983; Puškadija et al. 2007), hive weight (Hambleton 1925; Mitchener 1955; McLellan 1977; Atanasov et al. 2021) and flight activities (Lundie 1925; Szabo 1980; Burrill and Dietz 1981; Clarke and Robert 2018) are all very sensitive to the environmental factors such as the temperature and relative humidity, precipitation, wind speed, solar radiation, cloud cover, air pressure and even electrical disturbances (Lundie 1925) as well as air pollution (Thimmegowda et al. 2020; Cho et al. 2021; Jongh et al. 2022). However, there are differences in the sensitivity of different bee species (Vicens and Bosch 2000).

Atmospheric conditions impact bee activity and honey production through three ways. Short-term weather changes directly affect or can inhibit bee flight and foraging. Weather also affects foraging success indirectly through the biological response of nectar production of plants. Finally, long-term (seasonal) weather conditions largely determine phenology and the course of the blooming period of plants.

The honey production and the health of bee species directly influence human food security through pollination services (Gill et al. 2012; Rader et al. 2016). Since the early 1960s, crop yield has increased at an average annual growth rate of 1.5%, and our dependence on the honey bees increased accordingly (Aizen et al. 2008; Aizen and Harder 2009). Over the last five decades, the number of honey bee colonies increased by 45% globally, followed by a similar increase of honey production (Aizen and Harder 2009; Langowska et al. 2017). According to a more recent study, the global number of bee colonies nearly doubled, while global honey production tripled between 1961 and 2017 (Phiri et al. 2022). However, in Europe and North America, the number of colonies declined, while the honey production increased since 1961 (Phiri et al. 2022).

Studying the influence of weather on honey bees is not only important for the management of honey production, but also to better identify climate risks. In this review, existing research on short-term weather effects on bee behaviour and honey production are collected. In this sense, “short-term weather” is defined as weather phenomenae influencing bee flight on the intra-day timescale, including indirect weather impacts on plant physiology (e.g. nectar production). Species-dependent observations are reviewed primarily for two notable honey producing plants: black locust and sunflower. Table 1 summarizes the applied monitoring methods, the observed bee characteristics and atmospheric variables in the reviewed literature.

**Table 1:** An overview of selected studies of weather impacts on honey bee behaviour

Study	Methods					Observations				Atmospheric variables					Environment		
	Hive scale	Bee counter	Bee / swarming tracker	Camera / image processing	Manual / Lab obs.	Colony weight / honey prod.	Flight behaviour	Foraging behaviour	In-hive behaviour	Temperature	Radiation / sunshine dur.	Rain	Wind	Humidity	Location	Vegetation	Period
Mace (1912)	•					•				•	•	•	•	•	UK	diverse	April–July 1911
Hambleton (1925)	•					•				•	•			•	Somerset, USA	diverse	February–November 1922, May 1923
Lundie (1925)	•	•				•	•			•	•	•	•		Washington D. C., USA	diverse	April–July 1922
Mitchener (1955)	•					•					•	•	•		Manitoba, Canada	diverse	May–September 1924–1954
Szabo (1980)	•	•				•	•			•	•		•	•	Beaverlodge, Canada	diverse	July 1976 – July 1978
Burrill and Dietz (1981)		•					•			•	•			•	USA	diverse	April–May
Southwick and Moritz (1987)					•					•	•		•	•	New York, USA	NA	June–September
Vicens and Bosch (2000)				•	•		•			•	•	•	•	•	Girona, Spain	apple	1993–1995
Devillers et al. (2004)		•					•			•	•	•	•	•	Birieux, France	sunflower	July–September 2001
Campbell et al. (2005)		•					•	•		•					Bristol, Canada	blueberry	August 2003–2004
Freitas et al. (2007)					•		•					•			NE, Brazil	diverse	January–December 1999–2001
Puškadija et al. (2007)					•			•		•		•	•	•	Kneževi Vinogradi, Croatia	sunflower	2002
Meitalovs et al. (2009)				•					•	•				•	Jelgava, Latvia	NA	August-October 2008
Robertson et al. (2010)					•			•		•		•			New Zealand	tutu (*Coriaria arborea*)	January–February 2009
Rader et al. (2013)					•			•		•					New Jersey and eastern Pennsylvania, USA	watermelon	June–August 2005–2010
Brittain et al. (2013)					•			•					•		California, USA	almond	2008–2010
Chen et al. (2015)		•					•		•	•				•	New Taipei city, Taiwan	NA	July 2014
Lecocq et al. (2015)	•					•				•		•			Denmark	diverse	2010–2013
Cerrutti and Pontet (2016)					•			•		•		•			France	sunflower	2011–2013
He et al. (2016)			•				•			•		•		•	Nanchang, China	NA	NA
Jiang et al. (2016)		•					•		•	•		•		•	National Taiwan University	NA	March–April, July–September 2014
Meikle et al. (2016)	•			•		•			•	•					Arizona	NA	2013–2014
Shackleton et al. (2016)				•	•			•		•					São Paulo State, Brazil	diverse	February–March 2015
Gil-Lebrero et al. (2017)	•					•			•	•				•	Córdoba. Spain	sunflower	June–July 2016
Meikle et al. (2017)	•			•	•	•			•	•					USA	diverse	2014–2016
Pӑtruicӑ et al. (2017)	•					•				•		•		•	Romania	rape, acacia, sunflower	April–July 2017
Stalidzans et al. (2017)	•					•			•	•				•	Jelgava, Latvia	NA	2011, 2012 winter
Clarke and Robert (2018)		•					•	•		•	•	•	•	•	North Somerset, UK	NA	June–September 2013, July–September 2014
Meikle et al. (2018)	•			•		•				•		•			Arizona and California, USA, Sydney, Australia	diverse	2014, 2015–16, 2017
Henry et al. (2019)	acoustic sensors								•	•				•	Canada	NA	2015
Flores et al. (2019)	•					•				•		•		•	Córdoba, Spain	diverse	May–July 2016–2017
Hennessy et al. (2020)				•	•			•					•		Sussex, UK	NA	2017, 2018
Hong et al. (2020)	•	•				•	•			•				•	Gongchengzhen, China	NA	2018
Joshi and Joshi (2020)					•			•		•	•	•	•	•	Uttarakhand, India	apple	April–June 2019
Kviesis et al. (2020)	•					•				•				•	Jelgava, Latvia	NA	January–February 2020
Pӑtruicӑ et al. (2020)	•					•				•				•	Timiș county, Romania	sunflower	June–July 2018–2020
Rafael Braga et al. (2020)	•				•	•			•	•	•	•	•	•	USA	NA	January 2016 – December 2018
Solovev (2020)	•					•				•	•	•	•		Novgorod region, Russia	diverse	June–July 2018–2019
Atanasov et al. (2021)	•				•	•	•			•		•		•	Brestovica, Bulgaria	sunflower	2020
Hennessy et al. (2021)				•	•			•		•			•		Sussex, UK	lavender, marjoram	July 2018
Komasilova et al. (2021)	•					•				•		•	•	•	Latvia	NA	April–August
Ngo et al. (2021)				•			•	•		•	•	•	•	•	Hsinchu, Taiwan	NA	August–December 2019

## Methods for monitoring honey bee behaviour

The western honey bee (*Apis mellifera*) is a social insect that lives in colonies and whose main task is to survive and reproduce through foraging (Abou-Shaara 2014). In a colony of around 15,000–60,000 individuals, there are three casts of honey bees: worker bees, queen and drones (Southwick and Heldmaier 1987). The worker bees perform the most duties like the nectar, water and pollen foraging, therefore they are the most sensitive to external weather conditions (Kolmes et al. 1989). Worker activity depends on numerous factors such as the type of the pollinator species (Chacoff and Aizen 2006), colony size (Beekman et al. 2004) and health, the phenological phase and period (Couvillon et al. 2015; Danner et al. 2016), the landscape and the land use (Danner et al. 2016; Samuelson et al. 2022), the aim of the collection (Steffan-Dewenter and Kuhn 2003; Couvillon et al. 2015; Shackleton et al. 2016), the nectar source quantity and quality (Seeley 1986; Abrol 2006; Pasquale et al. 2013), the sex of the plants (Greenleaf and Kremen 2006), the distance from the nectar source (Steffan-Dewenter et al. 2002; Chacoff and Aizen 2006; Crane 2009), the foragers experience: “elite bees” (Klein et al. 2019), as well as environmental, weather conditions and the time of the day (Mace 1912; Lundie 1925; Farkas and Zajácz 2007; Puškadija et al. 2007; Clarke and Robert 2018).

Beekeepers have used visual inspection and weight measurement for decades to monitor the conditions in the hive (Hambleton 1925; McLellan 1977; Meikle et al. 2008; Meikle and Holst 2015). Agricultural development over the past decades opened new opportunities in studying bee behaviour (Meikle and Holst 2015; Zetterman 2018; Hong et al. 2020). Automatic hive scales with fine temporal resolution are often applied by beekeepers, and they provide important information of in-hive food reserves, nectar collection, signs of swarming and also provide an estimate of the number of workers (Zacepins et al. 2017; Meikle et al. 2018; Kviesis et al. 2020). Advanced devices exist for research applications. In-hive conditions can be monitored with thermometers (Zacepins 2013), acoustic sensors (Ferrari et al. 2008; Heise 2017) and vibration detection sensors (Bencsik et al. 2011). Bees exiting the hive are counted with capacitance sensors (Campbell et al. 2005), infrared sensors (Chen 2015), harmonic radar (Capaldi et al. 2000) or image processing methods (Chen et al. 2012; Magnier 2018; Ngo et al. 2021). Radio frequency identification (RFID) enable detailed bee flight tracking (Klein et al. 2019; Cho et al. 2021).

Special devices have also been developed for bee observation. *Apicard *can obtain the ingress and egress rate via electro-optical sensors at the hive entrance (Burrill and Dietz 1981). *ApiScan*,* Arnia*,* HiveMind *are systems based on counting the bees exiting the hive, as well as measuring external and hive temperature and humidity. Jiang et al. (2016) and Debauche et al. (2018) also developed complex systems for parallel observation of bee behaviour, external meteorological and in-hive conditions.

Recently, neural networks and automatic image processing systems have emerged to monitor honey bees (Boenisch 2018). Chen et al. (2012) developed an imaging system for monitoring and analysing the in-and-out activity of honey bees via circular character-encoding tags which were attached on the bees. Ngo et al. (2021) recorded the incoming and outgoing traffic at the entrance of five observed hives, then a deep learning-based classification algorithm isolated the pollen-carrying worker bees.

## Weather influence on bee flight activity

Local changes in meteorological factors such as air temperature, humidity, pressure, solar radiation intensity and wind velocity largely influence the behaviour of *Apis mellifera *L. (Southwick and Moritz 1987). Weather changes provoke instant response of flight activity (i.e., the number of bees entering and exiting the hives), or can inhibit flight entirely. The minimum temperature for bee flight is around 10 °C by most studies (Table 2), although bees may leave the hive occasionally at 4.5 °C (Heinrich 1996). Small fluctuations in the temperature are rapidly reflected in the number of bees entering and exiting the hives (Stone 1994; Vicens and Bosch 2000; Chen 2015; Razanova et al. 2021).

Vicens and Bosch (2000) found that *A. mellifera *is active when the air temperature is higher than 12–14 °C. During the experiment of Jiang et al. (2016), bees were more active when the daily mean ambient temperature was higher than 25 °C and the mean ambient relative humidity was between 60 and 70%. In the study by Razanova et al. (2021), the optimal temperature for flight was 20–25 °C, while Joshi and Joshi (2020) suggested that the optimum temperature should be higher than 16 °C. The upper limit of bee flight activity is near 40 °C (Heinrich 1979; Heinrich 1996) as reviewed by Abou-Shaara (2014) and Abou-Shaara et al. (2017). However, honey bees were found to fly in 46 °C (Heinrich 1996).

The classical work of Szabo (1980) highlighted the statistically significant positive correlation (R^2^= 0.28–0.52) between temperature and bee flight activity. Burrill and Dietz (1981) confirmed this result (R^2^ = 0.19–0.71) and extended with the confounding variables, solar radiation (R^2^ = 0.40–0.87) and relative humidity (R^2^= 0.65, negative correlation). According to Burrill and Dietz (1981), temperature fluctuations have a nearly linear effect on the activity of bees, while the response to radiation is more complicated. In the lower radiation range, the activity of bees changes linearly with the increase of the intensity, but above 460 W/m^2^, the overall activity decreases. Later studies found similar correlations with temperature and radiation (Devillers et al. 2004; R^2^= 0.62–0.72; Clarke and Robert 2018: R^2^ = 0.53–0.66), but the impact of wind and rain was also substantial.

Recent studies highlighted the negative effect of the air pollution on honey bees, however, the details of air pollution impacts on bee health and foraging are poorly understood due to the limited research. Thimmegowda et al. (2020) correlated the mechanistic effects of the air pollution (Particulate Matter - PM_10_) on the Giant Asian honey bee’s (*Apis dorsata*) health in Bangalore, India. They found significant survival reduction and physiological changes over 50 µg/m^3^ of PM_10_. Another finding claims that heavy pollution (PM_2.5_ mass concentration > 100 µg/m^3^) reduces the navigational ability of honey bees (*Apis mellifera*), leading to 30 min longer foraging trips, resulting in 71% more time spent with foraging than during pre-conditions (Cho et al. 2021) due to the limited orientation ability caused by the reduced visibility of the Sun’s position. Besides particulate matter, model simulations and in-situ measurements have also been carried out on the effects of other pollutants (near-surface ozone, hydroxyl radicals and nitrate radicals) on the spread of floral scent and insects foraging (Fuentes et al. 2016; Ryalls et al. 2022; Saunier et al. 2023). Their result was that the success rate of the insects reduced due to the suppression of the floral scent caused by air pollutants.

**Table 2:** A summary of weather limits and optimal conditions for honey bee flight

Reference	Minimum temperature	Maximum temperature	Optimal temperature	Optimal relative humidity	Influencing wind speed	Location	Period
Valló (1914)	10 °C		> 15 °C			Hungary	NA
Lundie (1925)	10 °C		16–25 °C		> 4.5 m/s	Washington DC, USA	April–July 1922
Örösi (1955)	10 °C					Hungary	NA
Burrill and Dietz (1981)	9 °C					USA	April–May
Heinrich (1979, 1996)	10 °C–4.5 °C for short periods	40–46 °C for short periods	17–25 °C			NA	NA
Jiang et al. (2016)			daily mean. > 25 °C	daily mean. 60–70%		National Taiwan University	March–April, July–September 2014
Abou-Shaara 2014 and Abou-Shaara et al. (2017)	10 °C–6.5 °C for short periods	40–46 °C for short periods	20 °C	75%		NA	NA
Razanova et al. (2021)	8 °C		17–32 °C		> 5–8 m/s	Vinnytsia, Ukraine	March–October

## Weather influence on foraging activity of honey bees

Foraging activity of honey bees (i.e. the number of visits on flowers and the duration of these flights) is correlated with flight activity, but it also depends on the availability and quality of the nectar, i.e., the phenological state of the plant and its short-term weather response affecting nectar production and sugar content (Farkas and Zajácz 2007). Therefore, weather limits and optimal conditions to foraging by honey bees (Table 3) are generally stricter than those obtained for flight activity, and strongly depend on the visited plant species. For example, in the study of Solovev (2020), honey collection stopped under 18 °C daily mean temperature. The minimum temperature for foraging black locust is 20 °C (Suhayda 1966; Farkas and Zajácz 2007). Rader et al. (2013) found the optimal temperature to be between 24 and 30 °C for foraging from watermelon flowers.

In the study of Ngo et al. (2021), the correlation of temperature, precipitation, wind and light intensity on collection behaviour were significant. In their calculations, the temperature (R^2^ = 0.25) and the light intensity (R^2^ = 0.08) were positively related, while rainfall (R^2^ = 0.36), humidity (R^2^ = 0.19) and wind speed (R^2^= 0.16) had a negative effect on the collection. The foraging duration is also significantly affected by the weather (He et al. 2016). Riessberger and Crailsheim (1997) showed that even short periods of rain and cold have a negative effect on foraging activity.

The influence of wind speed was observed by several studies (Pinzauti 1986). As the wind speed increases, honey bees tend to visit flowers less frequently and collect only from lower sections of the tree (Brittain et al. 2013). There seems to be a threshold velocity above which wind hampers foraging; this is estimated as > 4.5 m/s (Lundie 1925), > 5 m/s (Faluba 1983), > 2.5 m/s (Brittain et al. 2013), > 1.6–3.3 m/s (Ngo et al. 2021), > 5–8 m/s (Razanova et al. 2021). Honey bees can fly up to 2 km from the hive if necessary, but the distance heavily affects the collection efficiency (Crane 2009). Wind speed and direction (tailwind, headwind) not only affects the foraging success rate, but also the honey bees’ flight strategies (Burnett et al. 2020).

Hennessy et al. (2020, 2021) organized laboratory experiments to investigate wind influence on bee foraging. A wind speed of 2.8 m/s decreased the foraging rate by 37%, and there was a negative correlation between wind speed and the number of flowers successfully visited (R^2^= 0.055) (Hennessy et al. 2020). The wind also affected foraging distance negatively (R^2^= 0.43). In the next study of Hennessy et al. (2021), the increase of wind speed from 0 to 1 m/s to 2.5–3.5 m/s caused the decrease of the number of flowers visited by 36% for lavender and by 41% for marjoram.

**Table 3:** A summary of weather limits and optimal conditions for honey bee foraging

Reference	Minimum temperature	Maximum temperature	Optimal temperature	Optimal relative humidity	Influencing wind speed	Influencing precipitation	Location
Valló (1914)	15 °C						Hungary
Vicens and Bosch (2000)	12–14 °C (apple)						Girona, Spain
Suhayda (1966) Farkas and Zajácz (2007)	20 °C (black locust)						Hungary
Puškadija et al. (2007)			20–25 °C (sunflower)	65–70% (sunflower)			Kneževi Vinogradi, Croatia
Brittain et al. (2013)					> 5 m/s (almond)		California, USA
Rader et al. (2013)		40 °C	24–30 °C (watermelon)				New Jersey and eastern Pennsylvania, USA
Abou-Shaara (2014) and Abou-Shaara et al. (2017)	10 °C	40 °C					NA
He et al. (2016)						> 5 mm/day	Nanchang, China
Joshi and Joshi (2020)	10 °C (apple)		> 20 °C (apple)		> 6.7 m/s (apple)		Uttarakhand, India
Solovev (2020)	18 °C (daily mean)		> 24 °C (daily mean)			> 3 mm/day	Novgorod region, Russia
Ngo et al. (2021)					> 1.6–3.3 m/s		Hsinchu, Taiwan
Atanasov et al. (2021)			24–27 °C (sunflower)	54–59% (sunflower)			Brestovica, Bulgaria

## Weather influence on honey production

Foraging success is well represented by the easily measurable hive weight (i.e., bee and honey weight). However, differences among hives make it difficult to compare results obtained for different hives, and beekeeper operations must be accounted for. According to Holmes (2002), weather factors explain 80% of the variability of honey yields. The recent study by Atanasov et al. (2021) refined this estimate by examining colony weight during visits to sunflowers. They found positive correlation (R^2^ = 0.58) with air temperature, and negative correlation with the surface temperature of the sunflower blossom (R^2^ = 0.45), relative humidity (R^2^= 0.64). Monthly rainfall in June was found to explain 42% of variability in colony weight changes in a study by Lecocq et al. (2015). The relationship with rainfall in July was much weaker (R^2^ = 0.13), while the impact of temperature dominated (R^2^ = 0.15 in June and R^2^= 0.32 in July). Light rain had no influence on the weight gain. Moderate rain (4–8 mm/day) lowered the average daily weight gain by 60% (Solovev 2020). Bees were found to collect more efficiently before and after short rains (Devillers et al. 2004; He et al. 2016).

The main unifloral honeys in the European Union are the oilseed rape (*Brassica napus* L.), heather (*Calluna vulgaris* (L.) Hull), sweet chestnut (*Castanea sativa* Miller), citruses (*Citrus* spp), eucalyptuses (*Eucalyptus* spp.), sunflower (*Helianthus annuus* L.), lavenders (*Lavandula* spp.), alpenrose (Rhododendron spp.), black locust (*Robinia pseudoacacia* L.), rosemary (*Rosmarinus officinalis* L.), dandelion (*Taraxacum officinale* Weber), thymes (*Thymus* spp.) and lime trees (*Tilia *spp.) (Oddo et al. 2004). In this review, we selected for further discussion two important and frequently researched species, particularly in Central Europe: black locust (acacia) and sunflower.

### Honey production from black locust

Beekeepers expect excellent honey flow from black locust (*Robinia pseudoacacia *L.). However, honey yield is very variable, due to its high sensitivity on weather and hive conditions. Nectar production itself is influenced by numerous factors. According to the calculations of Keresztesi (1983), the average nectar production over 24 h is approximately 2 mg per flower. The honey yield by beehives can vary significantly. However, a single bee colony can produce up to 8 kg of acacia honey from black locust flowers (Samsonova et al. 2020). Other studies suggest that 1 hectare of black locust forest can yield between 159 and 1,000 kg of honey. It is estimated that a single beehive requires approximately 5.3 million flowers in a year to produce 87 kg of nectar, which is about 69 kg of honey (Carl et al. 2017). The sugar concentration varies between 44,8 ± 6,1% (Kim et al. 2023). The optimum conditions for nectar production are calm daytime temperatures of 20–25 °C (Márton 2011) or 16–25 °C (Pӑtruicӑ et al. 2017) and a moist environment with 80–90% relative humidity. However, black locust can already produce nectar when the air temperature reaches 10 °C (Pӑtruicӑ et al. 2017) or the ground temperature reaches 13–14 °C (Keresztesi 1983). Nectar secretion for black locust stops above 35 °C (Pӑtruicӑ et al. 2017). Practical observations suggest that warm nights (with temperatures above 15 °C) support black locust nectar production, and their flowers are extremely sensitive to late spring frost, which can cause zero honey production for beekeepers.

### Honey production from sunflower

Research on sunflower (*Helianthus annuus *L.) confirmed that the weather conditions, especially the precipitation and the temperature, as well as the selected hybrid significantly impact the nectar secretion, therefore the potential honey yield (Ion et al. 2008). Zajácz (2011) reported that different hybrids on different soil types produced varying amounts of nectar. According to beekeepers, 10–57 kg of sunflower honey can be obtained per beehive (Zajácz 2011; Takács and Oláh 2015). Mészáros and Gulyás (1994) observed that one disc floret produces nectar for 2 days. The assessable nectar mass from one disc floret of sunflower varied between 0.04 and 0.93 mg per floret in various measurements (Halmágyi and Suhayda 1963; Péter 1981; Zajácz et al. 2006; Ion et al. 2008; Chabert et al. 2020). One hectare of sunflower can yield 20–30 kg of honey (Lesznyák et al. 2007). Consequently, the potential sunflower honey yield varied between 6.1 and 52.6 kg/ha in different studies (Ion et al. 2008a; Ion et al. 2008b; Vlad et al. 2008; Kaur et al. 2020). However, optimal cultivation conditions can yield up to 250 kg of honey per hectare in the case of some hybrids (Bekić and Roljević 2013). The wide range of potential honey yield indicates its large sensitivity on the weather.

Experience has shown that sunflowers produce good honey when the average daytime temperature is between 22 and 24 °C, the relative humidity is between 50 and 80% and the soil has sufficient water (Márton 2011). Terzi (2017) reviewed 40 years of research on sunflower pollination in Serbia. Sunflower nectar secretion decreased above 27 °C and stopped completely at 33 °C. Similar upper temperature limit (35 °C) was found by Pӑtruicӑ et al. (2017). The greatest increase in colony weight was observed at daily temperatures of 24–27 °C, (Terzi 2017). Pӑtruicӑ et al. (2017) found a similar optimal temperature of 20–26 °C. In a 2002 study in Croatia, visitation was highest at 20–25 °C and 65–75% humidity (Puškadija et al. 2007). According to Atanasov et al. (2021), during the sunflower nectar collection, the optimal temperature is 24–27 °C, while the optimal humidity is 54–59%.

According to an analysis from Hungary between 2002 and 2004 (Zajácz 2011), the sugar content of nectar showed a moderate but statistically significant negative correlation with the humidity (R^2^ = 0.26), significant positive relationship with the temperature (R^2^= 0.19) and was also affected by the sunshine duration. Chabert et al. (2020) also found in France that air humidity regulates nectar sugar concentration and hence quantity. In their analysis, vapour pressure deficit explained 56% of the variation in sugar concentration. Nectar sugar concentration increased with temperature up to the optimum of 32 °C. Another study confirms that the mean maximum (R^2^ = 0.52) and mean minimum temperature (R^2^= 0.54) have statistically significant positive correlation, while the relative humidity has negative correlation with the nectar-sugar concentrations of sunflower (Kaur et al. 2020).

## Diurnal variability

**Fig. 1:**
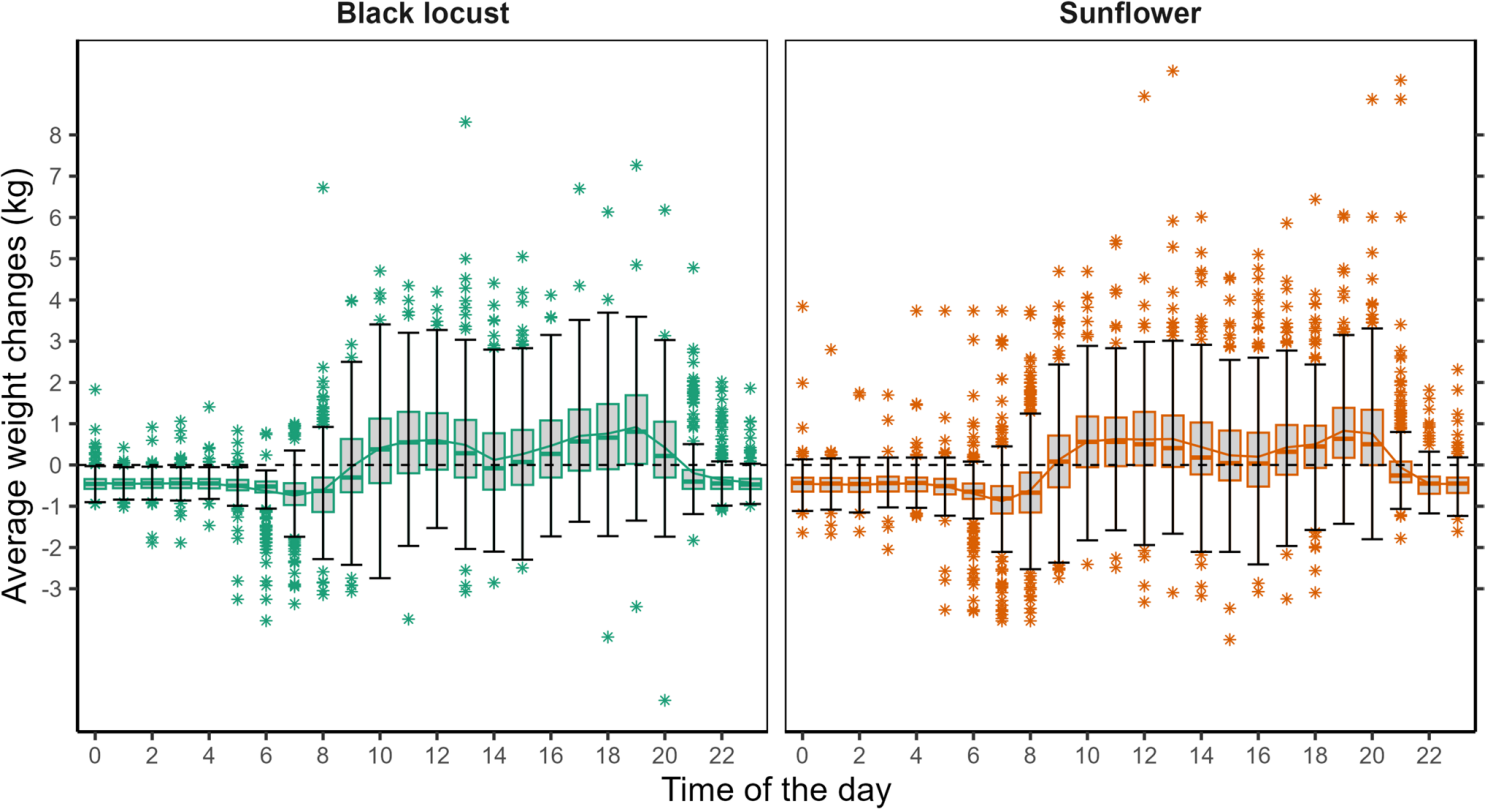
Standardized average hive weight change per hour of the day. Data source: hive scale measurements from the Hungarian apiarists’ network, 2020–2022

Bee flight has a characteristic diurnal cycle; therefore, its sensitivity on short-term weather phenomena depends on the time of day. The diurnal variability of bee flight follows the weather as well as the plant physiology, but it is also influenced by bee genetics. According to the classical literature of Szabo (1980), flight activity of honey bees in the summer began at around 9 AM local time, peaked between 2 and 4 PM and ended around 9 PM at Beaverlodge, Alberta, Canada. RFID tracking revealed that honey bee flight activity occurred between 5 and 21 h (Decourtye et al. 2011). However, more recent research showed peak activity in the late morning. Rader et al. (2013) found that the* Apis mellifera* was most active from 9 AM to 11 AM. Chen et al. (2012) and Jiang et al. (2016) found similar results: the flight activity started to rise at 5 AM (near sunrise), reached the peak at 10 AM and decreased until 7 PM (near sunset). A typical diurnal pattern of honey collection is presented in Fig. 1., obtained from multi-year averaging of hourly hive weight measurements.

The conflict between late morning and early afternoon peak activity can be explained by the fact that the diurnal variability of foraging is different according to the plant’s diurnal cycle for pollen and nectar production. In the study of Razanova et al. (2021), the maximum intensity of foraging from fruit trees was at 11 AM for pollen and at 1–2 PM for nectar. In case of black locust, the peak of pollen collection occurred from 9 AM to 1 PM, and the nectar collection increased from the morning to 2–3 PM. In contrast with the trees, foraging rate of sunflower pollen peaked as early as 9 AM, and bees did not collect sunflower pollen in the afternoon. Meanwhile, nectar collection from sunflower lasted from 9 to 19 h (Razanova et al. 2021). These results indicate that bees follow the plant’s diurnal variability of nectar and pollen production. Therefore, pollen foraging is extremely sensitive to short-term weather disturbances occurring during the late morning hours. Similarly, nectar gathering is particularly sensitive in the early afternoon.

## Response to the changing climate

Climate change is expected to be a significant concern for beekeeping and agriculture over the coming decades (Menzel et al. 2006; Hegland et al. 2009; Lever et al. 2014; Anderegg et al. 2021; Bezner Kerr et al. 2022). The latest IPCC report indicates a 49% extinction risk for insects due to the loss of climatically suitable habitats with a global temperature rise of around 3 °C (Parmesan et al. 2022). Jongh et al. (2022) reviewed research on climate change, pollutants, and antimicrobial resistance in relation to bee health. Insects are already responding to these changes (Deutsch et al. 2008). While warmer temperatures may extend the beekeeping season (Langowska et al. 2017), droughts and extreme weather can significantly reduce nectar production and hinder bee flight, threatening apiaries (Flores et al. 2019). Sparks et al. (2010) modeled that a 1 °C increase in average January–March temperatures could advance the first cleansing flight by about 4.4 ± 1.2 days. Other studies have found a weak correlation between the timing of the first cleansing flight and spring temperatures (Smoliński et al. 2021). Hegland et al. (2009) noted a linear relationship between the onset of plant flowering and the timing of the first pollinator flight in response to rising temperatures. Gordo and Sanz (2005) observed a quadratic change in the first appearance of *Apis mellifera *between 1943 and 2004.

Hegland et al. (2009) and Reddy et al. (2012) reviewed how rising temperatures affect plant-pollinator interactions, highlighting that pollination services are threatened by disruptions in flower-pollinator relationships, habitat changes, and food scarcity. Warming climate leads to advances in phenological events, creating a trophic mismatch between plants and pollinators (Thackeray et al. 2010; Bock et al. 2014; Marshall et al. 2023). Thackeray et al. (2010) studied phenological changes in 726 taxa in the UK, finding that terrestrial plants showed the fastest average phenological advances (5–6 days per decade), although only about half of the taxa exhibited significant trends, indicating potential trophic mismatches. Bock et al. (2014) found similar results. The phenological response of invertebrates was slower, advancing at approximately 4 days per decade on average (Thackeray et al. 2010). More recently, Marshall et al. (2023) highlighted an increasing risk of trophic mismatch between apple crops and their wild bee pollinators, particularly in Southern Europe. Using climate models, Chmielewski et al. (2005) projected phenophase advances of 3–27 days in Germany by 2050, raising concerns about trophic mismatches and the destabilization of plant-pollinator relationships.

## Discussion and conclusion

The impact of weather conditions on bee behaviour have been in the focus of research since the early 20th century. Besides seasonal weather influence on plant phenology, foraging by bees responds rapidly to short-term weather changes through direct (flight activity) and indirect (pollen and nectar production) effects. Understanding the short-range weather sensitivity of nectar and honey collection is important not only for the beekeepers, but for all agriculture depending on pollination services.

A classical research method is to use hive scales and establish a statistical relationship between colony weight or honey production and meteorological variables. Although this approach dates back to more than a century, recent applications also emerge using automatic hive scales with fine temporal resolution and detailed meteorological measurements. A more direct approach is to monitor bee flight behaviour with bee counters located at the hive exit. This method has evolved from manual counting and mechanical bee counters to electronic sensors, image processing and wireless networks. While the general approach is to observe the hives on field and couple the results with on-site meteorological measurements; there is also notable research which aims to create laboratory conditions for a controlled study of bee flight.

A wide range of studies confirm a significant positive relationship between air temperature and honey yield, and weather factors were found to explain 50–80% of variability in daily honey yields. The obtained statistical relationship between weather and honey yield involves direct (bee) and indirect (plant) weather response and is therefore dependent on the plant species.

Statistical correlations, critical and optimal values of temperature, wind, humidity, solar radiation and precipitation have been reviewed from numerous studies to describe the short-term (intra-day) weather impact on bee activity. However, the generalization of results is challenging due to differences in the foraged plant species, hive conditions and plant phenology. The short-term (intra-day) weather impacts on bee flight can be summarized into two primary aspects:

1. Direct obstruction of bee flight by weather: cold (< 10 °C) or warm (> 40 °C) temperature, wind (> 2–7 m/s), rain (> 3–5 mm/day) or haze. Honey collection is especially sensitive on obstructive weather in the late morning and early afternoon, when nectar production is maximal.
2. If flight is possible, bee activity follows nectar availability and quality, which is also influenced by the weather. Mild (20–30 °C) and moist (50–70%) midday weather is optimal for nectar production, but the details strongly depend on the plant species and its phenological stage.

Affordable devices (e.g. hive scales) and statistical methodology are available for beekeepers and agriculture to plan their production and adapt to weather-related risks. With the understanding of short-term weather impacts on bee activity, and exploiting the development of medium-range weather forecasts, beekeepers can better plan their honey production and mitigate weather risks on a daily timescale.
